# Mapping disparities in diabetic eye exam adherence using geographic information systems

**DOI:** 10.1371/journal.pone.0340804

**Published:** 2026-02-13

**Authors:** Neil Sai Dogra, Michael Patrick Geiss, Talia Gearinger, Alan Delmerico, Rajeev S. Ramchandran

**Affiliations:** 1 Department of Ophthalmology, Flaum Eye Institute, University of Rochester, Rochester, New York, United States of America; 2 Department of Public Administration, Maxwell School of Citizenship & Public Affairs, Syracuse University, Syracuse, New York, United States of America; 3 Department of Ophthalmology & Visual Sciences, SUNY Upstate Medical University, Syracuse, New York, United States of America; 4 Institute for Community Health Promotion, Buffalo State University, Buffalo, New York, United States of America; 5 Department of Public Health Sciences, University of Rochester, Rochester, New York, United States of America; 6 Center for Community Health and Prevention, University of Rochester, Rochester, New York, United States of America; South China University of Technology, CHINA

## Abstract

**Background:**

Diabetic retinopathy is a leading cause of preventable vision loss in adults, and timely retinal screening is essential for early detection and intervention. However, adherence to diabetic eye exam guidelines remains suboptimal, particularly in underserved populations. Geographic Information Systems (GIS) offer a novel approach to visualizing disparities in eye care access and adherence.

**Methods:**

We conducted a retrospective, cross-sectional study of 15,656 patients with diabetes mellitus (aged 18–75) receiving care in a university-based health system in Monroe County, NY, from November 2020 to November 2021. Eye exam adherence was determined using Healthcare Effectiveness Data and Information Set (HEDIS) criteria. Patient-level demographics and ZIP-code-level socioeconomic data were analyzed using ordinary least squares (OLS) regression. GIS choropleth maps were used to visualize regional variations in eye exam adherence and associated demographic and socioeconomic indicators.

**Results:**

Overall, 31.5% of patients were non-adherent to HEDIS eye exam standards. Non-adherence rates varied significantly by ZIP code (range: 13–50%) and were strongly associated with higher poverty (R² = 0.50, p < 0.0001), unemployment (R² = 0.17, p = 0.008), and lower educational attainment (R² = 0.50, p < 0.0001). Non-adherence also increased with higher proportions of Hispanic (R² = 0.24, p = 0.001) and non-Hispanic Black residents (R² = 0.45, p < 0.0001), and decreased with higher proportions of non-Hispanic White residents (R² = 0.45, p < 0.0001). GIS mapping identified an urban cluster of ZIP codes with consistently high non-adherence and socioeconomic risk profiles, as well as a rural outlier with high non-adherence but differing demographic characteristics.

**Conclusions:**

Our findings highlight geographic, socioeconomic, and racial disparities in diabetic eye exam adherence. GIS can serve as a powerful tool to identify high-risk populations and inform targeted outreach strategies aimed at reducing vision loss in vulnerable communities.

## Introduction

As of 2021, an estimated 38.1 million American adults had diabetes mellitus (DM), with prevalence increasing significantly over the previous two decades [1]. Roughly one in four DM patients develops diabetic retinopathy (DR), a microvascular retinal disease that poses the threat of irreversible vision loss through a variety of mechanisms, including retinal detachment, preretinal or vitreous hemorrhages, and macular edema [2]. DR is the leading cause of preventable blindness amongst the working-age population of developed countries [3,4].

Routine retinal screening is paramount in DM patients. Analyzing data from the 2005–2008 National Health and Nutrition Examination Survey, Gibson estimated that 73% of patients with DR were unaware of their condition [5]. Gibson noted that this is likely related to the fact that DR remains completely asymptomatic until late disease stages, at which point significant and permanent vision deficits can precipitously manifest [5] Without early detection of disease progression, it is often too late for interventions to be effective. The Healthcare Effectiveness Data and Information Set (HEDIS) Comprehensive Diabetes Care measures, which apply to adults aged 18–75 with DM, recommend retinal or dilated exams to be conducted by either an optometrist or ophthalmologist annually every two years for patients without DR, and annually for those with DR [6]. This standard is generally consistent with those of the American Academy of Ophthalmology and American Diabetes Association, which recommend retinal exams every year and two years respectively for DM patients [7,8]. However, in practice, DR screening adherence has shown to be markedly substandard, with rates of compliance to annual examinations ranging from 23–65% between studies [9–11]. Several socioeconomic and demographic factors have been reported to correlate with non-adherence, including lower income, nonwhite race, and fewer years of education.⁷ Given its association with dynamic community-level factors, eyecare utilization warrants continual monitoring as regions experience gradual changes in socioeconomic profile, as well as sudden, extreme disruptions such as those observed during the COVID-19 pandemic.

Geographic Information Systems (GIS) are computer systems that manage, display and analyze spatial data, allowing researchers to more easily identify geographic patterns. GIS mapping has become an increasingly powerful tool in visualizing access to care and identifying vulnerable patient populations [12–17]. But despite its potential in conducting spatial analyses of public health data and disparities, use of GIS in ophthalmologic epidemiology research has been notably limited [18]. With respect to DM and DR, GIS studies have largely centered around calculating driving times to eye care professionals [19–21]. and identifying regions with high disease prevalence [22–27]. Bryar et. al expanded upon one such analysis of DM and DR prevalence across Chicago area ZIP codes by identifying that high-risk ZIP codes had significantly more households identifying as ethnic minorities and/or living below the federal poverty line [28]. Jani et. al built off a similar GIS mapping of DR prevalence across ZIP codes in North Carolina by including plots of distance and driving time to professional eye care sites. However, this study only mapped patients already diagnosed with DR, which limited their GIS analysis to 361 patients and prevented them from visualizing DR rates against barriers to care [21].

Though GIS has effectively been used to examine spatial trends in DM and DR prevalence, it has not been utilized to thoroughly examine risk of disease progression. GIS has not yet been utilized in published literature to examine non-adherence rates in diabetic eye care, nor socioeconomic and demographic factors associated with these gaps. Better knowledge of these facets of eye care in those with diabetes is pivotal in identifying DM patient populations most vulnerable to irreversible vision loss from undetected or unmanaged disease progression. In the present analysis, we calculated rates of retinal eye exam non-adherence amongst DM patients from a university hospital system in Monroe County, New York, USA. We then utilized GIS alongside traditional statistical analyses to identify spatial, demographic, and socioeconomic factors associated with gaps in eye care for DM patients in a university-based health care system.

## Methods

### IRB statement

The University of Rochester Medical Center Institutional Review Board (Rochester, NY) waived the requirement for informed consent and approved this retrospective, cross-sectional analysis of de-identified patient data. All aspects of the project adhered to the tenets of the Declaration of Helsinki.

### Data collection & population criteria

We obtained de-identified patient data from a university-affiliated accountable care organization.

We included all adult DM patients (ages 18–75) with residence in Monroe County, NY who saw a provider in the University of Rochester Medical Center (URMC) Department of Internal Medicine from November 2020 to November 2021.

Measuring from the most recent primary care visit, we reviewed the time elapsed since each patient’s last retinal eye exam conducted by either an optometrist or ophthalmologist. DM patients with an eye exam negative for retinopathy within the previous two years or positive for retinopathy within one year were considered adherent to HEDIS standards for diabetic eye care.^6^ In addition to eye exam guideline adherence status, we recorded each patient’s residential ZIP code, age, and self-identified race/ethnicity from the electronic medical record.

Grouping by residential ZIP code, we calculated gap status—the proportion of patients non-adherent to HEDIS guidelines for diabetic eye care. To provide context about environmental socioeconomic metrics, we then consolidated the following publicly available ZIP-code-level data: racial and ethnic composition (% non-Hispanic Black, % non-Hispanic White, and % Hispanic residents), poverty, community unemployment, and education rate data from the 2021 American Community Survey conducted by the U.S. Census Bureau (ACS), diabetes long-term complications hospital admission rates from the NY State Department of Health’s 2020 Inpatient Prevention Quality Indicators, and DM prevalence rates from the Center for Disease Control’s Behavioral Risk Factor Surveillance System (BRFSS).

### Creation of GIS maps

We created a custom script in R Studio (version 4.3.0, R Foundation for Statistical Computing, Vienna, Austria) that merged our dataset of ZIP-code-level gap status, racial and ethnic proportions, socioeconomic indicators, and diabetic long-term complication rates with public domain TIGER/Line shapefiles of Monroe County, provided by the U.S. Census Bureau [29]. Across ZIP codes, we then used the ‘biscale’ statistical analysis package to calculate three equally sized tertiles (representing low, moderate, and high levels) for gap status and each demographic and socioeconomic predictor. We plotted univariate and bivariate choropleth maps to visualize eye exam gap status and its relationship with important factors across the ZIP Codes Monroe County, NY. For each choropleth map, ZIP codes were shaded according to the tertiles of the selected variable; darker shades corresponded to higher levels.

### Statistical analysis

We conducted linear Ordinary Least Square (OLS) regression models in R Studio (version 4.3.0, R Foundation for Statistical Computing, Vienna, Austria) to quantify the correlation between ZIP-code-level gap status and each of the specified demographic and socioeconomic predictors. Statistical significance was set at p < 0.05.

## Results

### Summary statistics

Of the 15,656 patients with DM and residence within Monroe County included in our cross-sectional analysis, mean age was 59 ± 12 years and 49.6% were women. Within our cohort, 31.5% of patients were not adherent to HEDIS standards for diabetic eye care (Table 1). Gap status, the proportion of patients not compliant with HEDIS diabetic eye care standards, ranged considerably between ZIP codes from 13% to 50%.

**Table 1:** Cohort demographics and summary statistics.

*Age*	Mean: 59 years, Std Dev: 12	Number	%
	18-44	2011	12.8
	45-64	7551	48.2
	≥65	6094	40.0
*Sex*			
	Male	7888	50.4
	Female	7768	49.6
*Race/Ethnicity*			
	Hispanic	987	6.3
	Non-Hispanic Black	3450	22.0
	Non-Hispanic White	10199	65.1
	Other	1020	6.5
*Eye Exam Status*			
	Open (non-compliant with HEDIS eye care standards)	4933	31.5
	Closed (compliant with HEDIS eye care standards)	10723	68.5

### Linear regressions

Using linear OLS regressions, ZIP-code-level gap status correlated significantly with higher long-term diabetic complication rates and DM prevalence within the adult population, higher rates of poverty and community unemployment, and higher proportions of residents without a high school diploma. Per ZIP code, gap status also increased significantly with higher proportions of Hispanic and non-Hispanic Black residents and lower proportions of non-Hispanic White residents (Table 2).

**Table 2:** OLS linear regressions.

Predictor	β	Standard Error	95% CI	R^2^	t	p
PQI Diabetic Long-term Complication Rate	0.0003	0.015	<0.0001 to 0.0005	0.16	2.67	0.012
BRFSS Prevalence of DM in Adult Population	0.009	0.003	0.003 to 0.01	0.22	3.29	0.002
ACS Poverty Rate	0.31	0.05	0.21 to 0.41	0.50	6.21	<0.0001
ACS Unemployment Rate	0.92	0.33	0.25 to 1.58	0.17	2.79	0.008
ACS Proportion of Residents Without a High School Diploma	0.50	0.08	0.34 to 0.67	0.50	6.19	<0.0001
ACS Proportion of Non-Hispanic Black Residents	0.20	0.04	0.13 to 0.28	0.45	5.73	<0.0001
ACS Proportion of Hispanic Residents	0.39	0.11	0.16 to 0.61	0.24	3.49	0.001
ACS Proportion of Non-Hispanic White Residents	−0.15	0.03	−0.21 to −0.10	0.45	−5.68	<0.0001

### GIS choropleth maps

**Fig 1:**
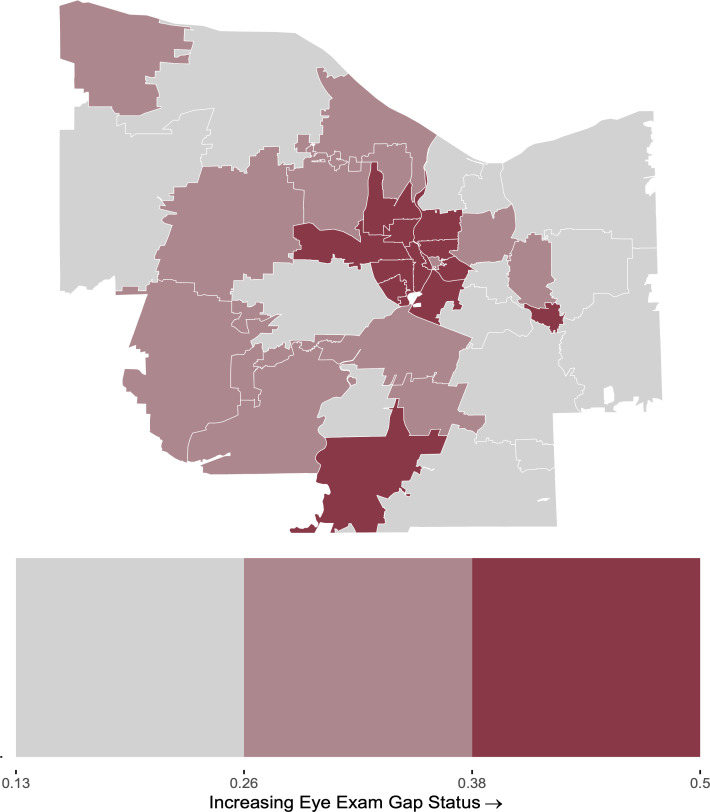
Choropleth map of gap status across ZIP codes in Monroe County. ZIP code boundaries were derived from public domain **U.**S. Census Bureau TIGER/Line shapefiles [29].

In a univariate choropleth plot of gap status across Monroe County, we identified a region of 11 central ZIP codes, which represent much of the city of Rochester, that were within the highest tertile of gap status. In contrast, peripheral ZIP codes of Monroe County, which represent suburbs of urban Rochester, displayed low or moderate gaps in diabetic eye care (Fig 1).

**Fig 2:**
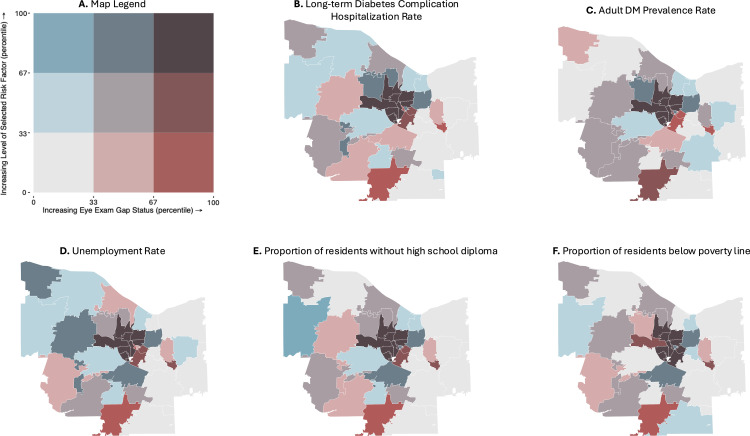
Bivariate choropleth maps of gap status against exogenous Monroe County ZIP-Code-Level socioeconomic predictors. ZIP code boundaries were derived from public domain **U.**S. Census Bureau TIGER/Line shapefiles [29]. **A.** Map legend, **B.** long-term diabetic complication rate, **C.** adult DM prevalence rate, **D.** community unemployment rate, **E.** proportion of residents without a high school diploma, and **F.** proportion of residents living below the poverty line.

**Fig 3:**
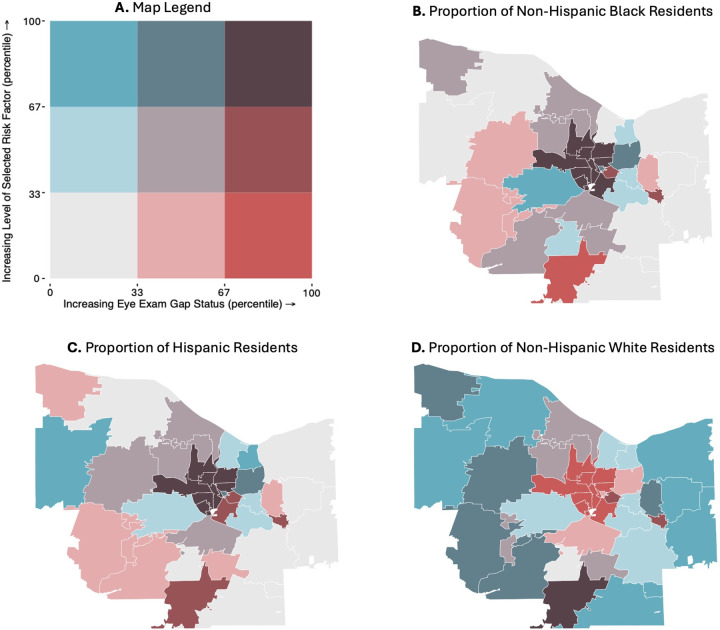
Bivariate choropleth maps of gap status against exogenous ZIP-Code-Level racial and ethnic composition. ZIP code boundaries were derived from public domain **U.**S. Census Bureau TIGER/Line shapefiles [29]. **A.** Map legend, **B.** proportion of non-Hispanic Black residents, **C.** proportion of Hispanic residents, and **D.** proportion of non-Hispanic White residents.

In bivariate choropleth maps of gap status and long-term diabetic complication rates and adult DM prevalence, the 11 central ZIP codes were within the highest tertile for both eye exam gap status and long-term inpatient diabetic complication rates, while ZIP codes in the periphery of Monroe County displayed low or moderate rates of both examined variables (Fig 2B,C). Similar results were observed in maps of gap status plotting eye exam gap status against socioeconomic indicators. The same cluster of central Monroe County ZIP codes in Rochester fell into the highest tertiles for proportions of residents (1) without employment, (2) without a high school diploma, and (3) living below the poverty line (Fig 2D-F). In maps of patient race and ethnicity, these same central ZIP codes with the highest gaps in timely eye care were among the lowest tertile for proportion of white patients and highest tertiles for proportions of Hispanic and Black patients. Conversely, peripheral ZIP codes generally displayed high proportions of white patients and low or moderate proportions of Hispanic and Black patients (Fig 3B-D).

One ZIP Code in the Southern edge of Monroe County, representing rural Rush, New York, also fell into the highest tertile of gap status (Fig 1). However, this ZIP code presented with socioeconomic and demographic qualities that were markedly distinct from the group of central ZIP codes with high gap status. Rush, NY fell into the lowest tertile for diabetic complication rates (Fig 2B) and for each socioeconomic predictor (Fig 2D-F). This ZIP code also displayed a moderate proportion of Hispanic patients, low proportion of non-Hispanic Black patients, and high proportion of non-Hispanic White patients (Fig 3B-D).

## Discussion

To the authors’ best knowledge, this is the first study to evaluate diabetic eye exam adherence rates using GIS and HEDIS primary care criteria. Our study included a sizable and diverse cohort, allowing for concurrent analysis of socioeconomic and demographic factors associated with gaps in diabetic eye care. Per HEDIS standards for diabetic eye care, our cohort displayed an average gap status across ZIP codes consistent with previously reported non-adherence rates, which range from 23–65% for annual retinal examinations [9–11]. However, gap status varied substantially between ZIP codes, with the lowest observed non-adherence rate of 13% falling considerably below the lower bound of previously reported non-adherence rates.

### OLS regressions of risk factors

Using OLS linear regressions, we found ZIP-code-level non-adherence rates within our cohort to be strongly correlated with a broad selection of public health, socioeconomic, and demographic factors. Gaps in timely eye care were significantly associated with higher population-level DM prevalence and higher rates of long-term complication hospital admissions secondary to a DM diagnosis. The strong association between these two population-level health metrics and gap status within our cohort suggests that regions with poor diabetic eye care may be burdened by holistically substandard DM disease management.

We also found higher eye exam gap status to be significantly associated with three indicators of low socioeconomic status from ZIP-code-level census data (community unemployment rate, low educational attainment, and poverty rate), which may reflect financial burdens and barriers associated with accessing routine ophthalmic care. This finding corroborates established socioeconomic disparities in diabetic eye care, with previous studies finding that socioeconomically disadvantaged DM patients are at a higher risk of developing DR, receiving a more severe DR diagnosis when first diagnosed, and displaying substandard adherence to disease management post-diagnosis [30–32]. Access to routine DR screening has also previously been associated with insurance status, with patients covered by Medicaid finding less success in obtaining eye care appointments than those with private insurance [30]. Expanding on our correlations with census data and existing literature, future studies should analyze how diabetic eye care varies with insurance status within our university health system.

Lastly, we correlated gap status with community-level racial and ethnic composition. Racial and ethnic composition within ZIP codes were significantly associated with diabetic eye exam adherence. We found that ZIP codes with more minoritized populations, characterized by relatively higher proportions of Hispanic and non-Hispanic Black residents and lower proportions of non-Hispanic White residents, presented with significantly higher gaps in diabetic eye care. Our findings from OLS regressions corroborate recognized racial and ethnic disparities in DM eye care, which include greater delays in accessing care and higher risks of developing proliferative DR among minoritized patient populations [7,33,34].

### GIS mapping of gap status against identified risk factors

Upon identifying several socioeconomic and demographic risk factors associated with gaps in diabetic eye care, we utilized GIS to discern spatial relationships between these measures and locate the regions most impacted by these inequities. Mapping gap status across Monroe County, we identified a group of 11 central ZIP codes, representing the city of Rochester, that fell into the highest tertile of gaps in timely eye care. In bivariate choropleth maps, these same ZIP codes fell into the most extreme tertile for each significant risk factor from OLS regressions: high rates of DM prevalence and long-term diabetic complications, low socioeconomic status, low proportions of non-Hispanic White residents, and high proportions of Hispanic and non-Hispanic Black residents. In contrast, suburban ZIP codes in the periphery of Monroe County generally displayed low or moderate gap statuses, as well as low and moderate levels of each socioeconomic and demographic risk factor.

Thus, GIS mapping of eye exam gaps against identified risk factors was consistent with trends observed in our OLS regressions. Our findings suggest that urban regions may be more susceptible to gaps in diabetic eye care than nearby suburbs; these disparities may reflect the considerable differences in overall DM disease management, socioeconomic status, and racial/ethnic composition between urban and suburban regions. GIS mapping allowed us to effectively identify the region of Monroe County most impacted by gaps in timely diabetic eye care and contextualize its socioeconomic and demographic landscape, which we found to be strongly associated with identified disparities in care.

With GIS mapping, we also identified one ZIP code on the Southern edge of Monroe County that fell into the highest tertile of gap status, despite showing a socioeconomic and demographic landscape that was markedly deviant from previously discussed patterns. This ZIP code, which represented rural Rush, NY, presented with low rates of long-term diabetic complications, high socioeconomic status, and a high proportion of white patients. That Rush, NY still fell into the highest tertile of gap status indicates that rural communities may face a distinct combination of barriers that limit access to care, such as larger distances to eye clinics or substandard health literacy [31]. However, because these data were derived from patients seen within a single health system, they may not be fully representative of the broader population, which may include patients who receive care from other nearby health systems. This potential limitation underscores the need for further investigation into cross-system patterns of care delivery. This finding further highlights a need for future research examining diabetic care and risk factors, such as travel burden, which may be associated with rural patient populations within our hospital system. Moreover, as an outlier to previously discussed trends in barriers to DM eye care, this ZIP code exemplifies the utility of GIS in pinpointing vulnerable regions that may go undetected in analyses of risk factors alone.

### Strengths

Our study has several strengths. We include a diverse and sizable cohort from a university hospital system, as well as several exogenous ZIP-code-level metrics, allowing us to examine a broad range of risk factors. Leveraging GIS in combination with traditional statistical analyses, we successfully identified several socioeconomic, demographic, and spatial risk factors associated with gaps in diabetic eye care, as well as regions that deviated from observed patterns.

### Limitations

This study has several limitations that should be considered when interpreting its findings. Analyses were conducted using ZIP-code level predictors and univariate modeling. Thus, associations are intended to be interpreted at the community level, rather than as predictors of individual-level risk, and do not reflect causality. Further investigation is needed to elucidate how patient-level factors, including insurance status and transportation access, predict eye care utilization within our health system. Despite these limitations, this analytic framework offered distinct strengths. ZIP-code aggregation enabled consistent linkage of electronic health record data with publicly available socioeconomic indicators, allowing for the construction of a sizable and demographically diverse dataset that captured region-wide trends to inform targeted outreach at the community level.

Secondly, our data source is limited to a single university health system, which is one of two primary hospital systems in the Western New York and Finger Lakes region [35]. Thus, our cohort does not encompass all patients aged 18–75 with DM in Monroe County, and our calculated gap statuses may not accurately reflect non-adherence rates for ZIP codes where a substantial portion of patients utilize a separate health network for their primary care. Importantly, gap status was derived from insurance claims and thus remains a valid metric, even for patients who received eyecare outside of our health system. Still, this restriction introduces potential selection bias at the geographic level, as certain ZIP codes may contain large populations whose primary care occurs outside our network. Consequently, observed differences in adherence between ZIP codes may partially reflect differences in health-system market share or referral patterns rather than true variation in patient behavior. Future work comparing eye care utilization across health systems and against census-based denominators will clarify representativeness and validate these regional estimates.

Lastly, we acknowledge that our diabetic non-adherence rates may contain unique bias due to the considerable changes in healthcare service delivery related to the COVID-19 pandemic, as patients’ diabetic eye exam status was determined from appointment status in November 2020 to November 2021. For example, it was more difficult for DM patients to access primary care providers, specialty physicians, and ophthalmologists due to the COVID-19 pandemic during 2020 and 2021 that produced extensive healthcare clinic restrictions that significantly impeded vision care access. Still, overall rates of nonadherence fell within previously published ranges [9–11]. Moreover, community-level healthcare utilization patterns during this pandemic warrant particular attention as we examine the extent of disparities in access to care within our health system. Our findings that race, ethnicity, and socioeconomic status were strongly associated with diabetic care gaps during an unprecedented pandemic may reflect underlying vulnerabilities within our health system and serve as a valuable reference as clinicians, administrators, and policymakers evaluate and define post-pandemic healthcare delivery.

## Conclusion

Frequency of diabetic eye exams falls significantly below recommended standards and varies considerably within the DM patient population. GIS mapping of regional ophthalmic data can help providers identify geographic, socioeconomic and demographic characteristics of DM patients vulnerable to vision loss from gaps in timely eye care. These insights may inform more targeted outreach efforts in specific neighborhoods with relationship building with community liaisons and resources to address care gaps and prevent disease progression. More research is needed to explore how diabetic eye gaps vary amongst rural, suburban, and urban communities and underinsured DM patient populations, as well as the clinical effectiveness of GIS-driven interventions in improving patient outcomes.

## Supporting information

S1 Table Full analytic dataset used in presented analyses of diabetic eye exam adherence. The file is provided in Microsoft Excel format.
(DOCX)

## References

[1] Center for Disease Control. National diabetes statistics report. 2024.

[2] Lundeen EA, Burke-Conte Z, Rein DB, Wittenborn JS, Saaddine J, Lee AY, et al. Prevalence of Diabetic Retinopathy in the US in 2021. JAMA Ophthalmol. 2023;141(8):747–54. doi: 10.1001/jamaophthalmol.2023.2289 37318810 PMC10273133

[3] Porta M, Bandello F. Diabetic retinopathyA clinical update. Diabetologia. 2002;45(12):1617–34. doi: 10.1007/s00125-002-0990-7 12488951

[4] Fang L, Sheng H, Tan Y, Zhang Q. Prevalence of diabetes in the USA from the perspective of demographic characteristics, physical indicators and living habits based on NHANES 2009-2018. Front Endocrinol (Lausanne). 2023;14:1088882. doi: 10.3389/fendo.2023.1088882 36960397 PMC10028205

[5] Gibson DM. Diabetic retinopathy and age-related macular degeneration in the U.S. Am J Prev Med. 2012;43(1):48–54. doi: 10.1016/j.amepre.2012.02.028 22704745

[6] Eye exam for patients with diabetes. 2024.

[7] An J, Niu F, Turpcu A, Rajput Y, Cheetham TC. Adherence to the American Diabetes Association retinal screening guidelines for population with diabetes in the United States. Ophthalmic Epidemiol. 2018;25(3):257–65. doi: 10.1080/09286586.2018.1424344 29333897

[8] Flaxel CJ, Adelman RA, Bailey ST, Fawzi A, Lim JI, Vemulakonda GA, et al. Diabetic Retinopathy Preferred Practice Pattern®. Ophthalmology. 2020;127(1):P66–145. doi: 10.1016/j.ophtha.2019.09.025 31757498

[9] Eppley SE, Mansberger SL, Ramanathan S, Lowry EA. Characteristics Associated with Adherence to Annual Dilated Eye Examinations among US Patients with Diagnosed Diabetes. Ophthalmology. 2019;126(11):1492–9. doi: 10.1016/j.ophtha.2019.05.033 31281055

[10] Paksin-Hall A, Dent ML, Dong F, Ablah E. Factors contributing to diabetes patients not receiving annual dilated eye examinations. Ophthalmic Epidemiol. 2013;20(5):281–7. doi: 10.3109/09286586.2013.789531 23662945

[11] Shi Q, Zhao Y, Fonseca V, Krousel-Wood M, Shi L. Racial disparity of eye examinations among the U.S. working-age population with diabetes: 2002-2009. Diabetes Care. 2014;37(5):1321–8. doi: 10.2337/dc13-1038 24574354 PMC4876755

[12] Davis J, Liu M, Kao D, Gu X, Cherry-Peppers G. Using GIS to Analyze Inequality in Access to Dental Care in the District of Columbia. AMA J Ethics. 2022;24(1):E41-47. doi: 10.1001/amajethics.2022.41 35133727 PMC9210465

[13] Sanchez FG, Gardiner SK, Demirel S, Rees JP, Mansberger SL. Geospatial analysis of blindness within rural and urban counties. PLoS One. 2022;17(10):e0275807. doi: 10.1371/journal.pone.0275807 36215279 PMC9550029

[14] Chen KW, Jiang A, Kapoor C, Fine JR, Brandt JD, Chen J. Geographic Information System Mapping of Social Risk Factors and Patient Outcomes of Pediatric Glaucoma. Ophthalmol Glaucoma. 2023;6(3):300–7. doi: 10.1016/j.ogla.2022.10.008 36427749

[15] McLafferty SL. GIS and health care. Annu Rev Public Health. 2003;24:25–42. doi: 10.1146/annurev.publhealth.24.012902.141012 12668754

[16] McLafferty S, Grady S. Prenatal care need and access: a GIS analysis. J Med Syst. 2004;28(3):321–33. doi: 10.1023/b:joms.0000032848.76032.28 15446621

[17] Stansberry TT, Tran L, Myers C. Using Geographic Information Systems in health disparities research: Access to care considerations. Res Nurs Health. 2023;46(6):635–44. doi: 10.1002/nur.22348 37840372

[18] Soares RR. The evolving field of Big Data: understanding geographic information systems analysis and its transformative potential in ophthalmic research. Curr Opin Ophthalmol. 2022;33(3):188–94. doi: 10.1097/ICU.0000000000000839 35220329

[19] Shaffer J, Rajesh A, Stewart MW, Lee AY, Miller DD, Lee CS, et al. Evaluating Access to Laser Eye Surgery by Driving Times Using Medicare Data and Geographical Mapping. JAMA Ophthalmol. 2023;141(8):776–83. doi: 10.1001/jamaophthalmol.2023.3061 37471084 PMC10360006

[20] Stein JD, Kapoor KG, Tootoo JL, Li R, Wagner A, Andrews C, et al. Access to Ophthalmologists in States Where Optometrists Have Expanded Scope of Practice. JAMA Ophthalmol. 2018;136(1):39–45. doi: 10.1001/jamaophthalmol.2017.5081 29167903 PMC5833600

[21] Jani PD, Forbes L, McDaniel P, Viera A, Garg S. Geographic Information Systems Mapping of Diabetic Retinopathy in an Ocular Telemedicine Network. JAMA Ophthalmol. 2017;135(7):715–21. doi: 10.1001/jamaophthalmol.2017.1153 28520876 PMC5710206

[22] Nurjannah N, Baker KM. Using GIS and death records to inform statewide school-based diabetes prevention interventions in Michigan. J Public Health Res. 2020;9(4):1887. doi: 10.4081/jphr.2020.1887 33381471 PMC7750886

[23] Kruger DJ, Brady JS, Shirey LA. Using GIS to facilitate community-based public health planning of diabetes intervention efforts. Health Promot Pract. 2008;9(1):76–81. doi: 10.1177/1524839906293396 17494946

[24] Curtis AB, Kothari C, Paul R, Connors E. Using GIS and secondary data to target diabetes-related public health efforts. Public Health Rep. 2013;128(3):212–20. doi: 10.1177/003335491312800311 23633736 PMC3610073

[25] Faka A, Chalkias C, Montano D, Georgousopoulou EN, Tripitsidis A, Koloverou E, et al. Association of Socio-Environmental Determinants with Diabetes Prevalence in the Athens Metropolitan Area, Greece: A Spatial Analysis. Rev Diabet Stud. 2018;14(4):381–9. doi: 10.1900/RDS.2017.14.381 29590231 PMC6230444

[26] Kameel M, Steven S, Aleem K, Dayna L, Demeytri R, Shanala S, et al. Evaluation and Use of Registry Data in a GIS Analysis of Diabetes. AIMS Public Health. 2015;2(3):318–31. doi: 10.3934/publichealth.2015.3.318 29546113 PMC5690238

[27] Curtis AJ, Lee W-AA. Spatial patterns of diabetes related health problems for vulnerable populations in Los Angeles. Int J Health Geogr. 2010;9:43. doi: 10.1186/1476-072X-9-43 20796322 PMC2939634

[28] Bryar PJ, Wang A, Eichinger SE, Agron S, Langguth A, Mbagwu M, et al. Health Care Disparities in Diabetes and Diabetic Retinopathy. Ophthalmic Epidemiol. 2023;30(5):453–61. doi: 10.1080/09286586.2023.2168015 36705505 PMC10372196

[29] Bureau USC. Data from: TIGER/Line Shapefile, Current, County, Monroe County, NY, Address Ranges Relationship File. 2024.

[30] Patel D, Ananthakrishnan A, Lin T, Channa R, Liu TYA, Wolf RM. Social Determinants of Health and Impact on Screening, Prevalence, and Management of Diabetic Retinopathy in Adults: A Narrative Review. J Clin Med. 2022;11(23):7120. doi: 10.3390/jcm11237120 36498694 PMC9739502

[31] Peavey JJ, D’Amico SL, Kim BY, Higgins ST, Friedman DS, Brady CJ. Impact of Socioeconomic Disadvantage and Diabetic Retinopathy Severity on Poor Ophthalmic Follow-Up in a Rural Vermont and New York Population. Clin Ophthalmol. 2020;14:2397–403. doi: 10.2147/OPTH.S258270 32904606 PMC7457718

[32] Guo Y, Copado IA, Yonamine S, Jian Ma C, McLeod S, Arnold BF, et al. The Relationship Between Health Insurance Status and Diabetic Retinopathy Progression. Ophthalmol Sci. 2023;4(3):100458. doi: 10.1016/j.xops.2023.100458 38317868 PMC10840328

[33] Huang BB, Radha Saseendrakumar B, Delavar A, Baxter SL. Racial Disparities in Barriers to Care for Patients With Diabetic Retinopathy in a Nationwide Cohort. Transl Vis Sci Technol. 2023;12(3):14. doi: 10.1167/tvst.12.3.14 36928128 PMC10029769

[34] Chan AX, McDermott Iv JJ, Lee TC, Ye GY, Shahrvini B, Radha Saseendrakumar B, et al. Associations between healthcare utilization and access and diabetic retinopathy complications using All of Us nationwide survey data. PLoS One. 2022;17(6):e0269231. doi: 10.1371/journal.pone.0269231 35704625 PMC9200294

[35] Monroe County Department of Public Health. Monroe County Joint Community Health Needs Assessment. 2024.

