# Supplementary material for: Mapping disparities in diabetic eye exam adherence using geographic information systems
Source: PLoS One. 2026 Feb 13;21(2):e0340804. doi: 10.1371/journal.pone.0340804 (PMC12904463; doi:10.1371/journal.pone.0340804)
Supplement: S1 Table — The file is provided in Microsoft Excel format. (DOCX) [file pone.0340804.s001.docx]

| ZipCode | City | County | Total Population | % Hispanic | % Non-Hispanic White | % Non-Hispanic Black | % Without High School Diploma | % Unemployed | % Below Poverty Line | DM Prevalence | PQI Diabetes Long-Term Complication Rate | # Patients | Average # Months from eye exam | Gap Status (%) |
| --- | --- | --- | --- | --- | --- | --- | --- | --- | --- | --- | --- | --- | --- | --- |
| 14420 | Brockport | Monroe | 20955 | 7% | 88% | 2% | 9% | 4% | 16% | 8.1 | 76.56 | 207 | 15.45026 | 28% |
| 14428 | Churchville | Monroe | 7814 | 2% | 96% | 1% | 6% | 2% | 7% | 9.3 | 108.93 | 130 | 15.90984 | 32% |
| 14445 | East Rochester | Monroe | 8006 | 5% | 84% | 6% | 6% | 4% | 14% | 9.1 | 55.86 | 221 | 17.77949 | 38% |
| 14450 | Fairport | Monroe | 41298 | 3% | 90% | 2% | 4% | 2% | 5% | 8.9 | 51.12 | 1016 | 14.25941 | 24% |
| 14464 | Hamlin | Monroe | 7614 | 2% | 93% | 4% | 7% | 4% | 9% | 9 | 88.76 | 82 | 17.17333 | 32% |
| 14467 | Henrietta | Monroe | 10413 | 3% | 76% | 9% | 8% | 3% | 14% | 10.2 | 110.29 | 365 | 15.79878 | 30% |
| 14468 | Hilton | Monroe | 18320 | 4% | 94% | 1% | 4% | 4% | 5% | 9.1 | 86.38 | 254 | 14.97854 | 26% |
| 14472 | Honeoye Falls | Monroe | 8297 | 1% | 95% | 1% | 3% | 2% | 9% | 9 | 14.32 | 125 | 14.55263 | 26% |
| 14506 | Mendon | Monroe | 1468 | 0% | 99% | 0% | 1% | 2% | 1% | 8.6 | 96.34 | 15 | 13.06667 | 13% |
| 14514 | North Chili | Monroe | 6812 | 4% | 87% | 3% | 6% | 7% | 9% | 9.3 | 166.54 | 94 | 16.22353 | 32% |
| 14526 | Penfield | Monroe | 20662 | 4% | 88% | 3% | 5% | 3% | 4% | 9.3 | 42.47 | 459 | 14.29952 | 30% |
| 14534 | Pittsford | Monroe | 31596 | 2% | 87% | 2% | 2% | 2% | 2% | 9.4 | 11.98 | 617 | 14.98785 | 25% |
| 14543 | Rush | Monroe | 3285 | 7% | 91% | 2% | 3% | 1% | 5% | 9.4 | 0 | 115 | 16.87736 | 37% |
| 14546 | Scottsville | Monroe | 4529 | 4% | 89% | 5% | 5% | 3% | 13% | 9.8 | 74.98 | 147 | 17.06569 | 32% |
| 14559 | Spencerport | Monroe | 18687 | 7% | 91% | 1% | 4% | 5% | 8% | 9.4 | 62.85 | 224 | 16.51961 | 30% |
| 14580 | Webster | Monroe | 51917 | 3% | 88% | 3% | 5% | 3% | 5% | 9.1 | 74.01 | 1100 | 14.77429 | 27% |
| 14586 | West Henrietta | Monroe | 11691 | 4% | 72% | 7% | 7% | 3% | 8% | 8.2 | 79.73 | 402 | 16.19137 | 30% |
| 14604 | Rochester | Monroe | 1932 | 10% | 48% | 32% | 20% | 3% | 41% | 16.1 | 230.84 | 74 | 15.14063 | 32% |
| 14605 | Rochester | Monroe | 13106 | 35% | 11% | 49% | 30% | 11% | 48% | 18.9 | 194.6 | 230 | 19.10294 | 37% |
| 14606 | Rochester | Monroe | 27890 | 14% | 57% | 22% | 20% | 5% | 20% | 12.8 | 210.1 | 638 | 17.35879 | 37% |
| 14607 | Rochester | Monroe | 16818 | 5% | 80% | 11% | 6% | 4% | 23% | 6.8 | 76.52 | 244 | 16.92488 | 38% |
| 14608 | Rochester | Monroe | 12340 | 10% | 19% | 61% | 24% | 11% | 48% | 18.3 | 245.16 | 309 | 17.68727 | 39% |
| 14609 | Rochester | Monroe | 42860 | 16% | 46% | 33% | 15% | 6% | 23% | 12.1 | 160.76 | 1015 | 16.81502 | 34% |
| 14610 | Rochester | Monroe | 14031 | 6% | 83% | 5% | 6% | 2% | 9% | 9.2 | 35.12 | 278 | 15.02672 | 29% |
| 14611 | Rochester | Monroe | 17169 | 11% | 21% | 63% | 25% | 9% | 42% | 19 | 238.57 | 502 | 18.15743 | 38% |
| 14612 | Rochester | Monroe | 34118 | 6% | 84% | 5% | 8% | 3% | 9% | 10.3 | 85.76 | 667 | 16.75163 | 30% |
| 14613 | Rochester | Monroe | 13380 | 22% | 28% | 38% | 21% | 8% | 28% | 15.3 | 382.83 | 270 | 20.05932 | 44% |
| 14614 | Rochester | Monroe | 1218 | 14% | 27% | 59% | 36% | 1% | 52% | 10.7 | 0 | 8 | 24 | 50% |
| 14615 | Rochester | Monroe | 16330 | 13% | 58% | 22% | 15% | 6% | 23% | 11.6 | 157.62 | 352 | 16.94081 | 35% |
| 14616 | Rochester | Monroe | 27605 | 6% | 80% | 9% | 8% | 3% | 10% | 10.7 | 122.96 | 534 | 15.29202 | 34% |
| 14617 | Rochester | Monroe | 22832 | 6% | 87% | 4% | 6% | 3% | 6% | 9.7 | 90.81 | 381 | 15.42857 | 25% |
| 14618 | Rochester | Monroe | 22955 | 4% | 82% | 4% | 2% | 2% | 8% | 8.5 | 39.65 | 455 | 14.75426 | 29% |
| 14619 | Rochester | Monroe | 14353 | 8% | 17% | 71% | 15% | 9% | 28% | 15.8 | 224.26 | 440 | 17.58639 | 38% |
| 14620 | Rochester | Monroe | 24519 | 7% | 68% | 14% | 8% | 4% | 22% | 8.9 | 82.75 | 487 | 16.35499 | 36% |
| 14621 | Rochester | Monroe | 33814 | 36% | 17% | 43% | 30% | 11% | 41% | 18.6 | 214.87 | 698 | 16.78846 | 34% |
| 14622 | Rochester | Monroe | 12060 | 12% | 82% | 5% | 8% | 2% | 10% | 10.7 | 113.18 | 284 | 15.59533 | 29% |
| 14623 | Rochester | Monroe | 25952 | 5% | 69% | 10% | 13% | 5% | 21% | 7.4 | 51.97 | 597 | 16.06691 | 30% |
| 14624 | Rochester | Monroe | 36265 | 5% | 80% | 12% | 7% | 4% | 6% | 9.6 | 82.98 | 816 | 15.40052 | 28% |
| 14625 | Rochester | Monroe | 10026 | 3% | 94% | 1% | 6% | 2% | 5% | 9.9 | 58.18 | 212 | 16.43523 | 32% |
| 14626 | Rochester | Monroe | 30769 | 5% | 83% | 6% | 8% | 3% | 7% | 11 | 131.59 | 571 | 16.26386 | 32% |

**S1 Table. Full analytic dataset**
